# Pruritus: A Sensory Symptom Generated in Cutaneous Immuno-Neuronal Crosstalk

**DOI:** 10.3389/fphar.2022.745658

**Published:** 2022-03-07

**Authors:** Attila Gábor Szöllősi, Attila Oláh, Erika Lisztes, Zoltán Griger, Balázs István Tóth

**Affiliations:** ^1^ Department of Immunology, Faculty of Medicine, University of Debrecen, Debrecen, Hungary; ^2^ Department of Physiology, Faculty of Medicine, University of Debrecen, Debrecen, Hungary; ^3^ Division of Clinical Immunology, Department of Internal Medicine, Faculty of Medicine, University of Debrecen, Debrecen, Hungary

**Keywords:** itch, molecular transduction of pruritus, sensory neurons, inflammation, skin, cytokines, dermatoses

## Abstract

Pruritus or itch generated in the skin is one of the most widespread symptoms associated with various dermatological and systemic (immunological) conditions. Although many details about the molecular mechanisms of the development of both acute and chronic itch were uncovered in the last 2 decades, our understanding is still incomplete and the clinical management of pruritic conditions is one of the biggest challenges in daily dermatological practice. Recent research revealed molecular interactions between pruriceptive sensory neurons and surrounding cutaneous cell types including keratinocytes, as well as resident and transient cells of innate and adaptive immunity. Especially in inflammatory conditions, these cutaneous cells can produce various mediators, which can contribute to the excitation of pruriceptive sensory fibers resulting in itch sensation. There also exists significant communication in the opposite direction: sensory neurons can release mediators that maintain an inflamed, pruritic tissue-environment. In this review, we summarize the current knowledge about the sensory transduction of pruritus detailing the local intercellular interactions that generate itch. We especially emphasize the role of various pruritic mediators in the bidirectional crosstalk between cutaneous non-neuronal cells and sensory fibers. We also list various dermatoses and immunological conditions associated with itch, and discuss the potential immune-neuronal interactions promoting the development of pruritus in the particular diseases. These data may unveil putative new targets for antipruritic pharmacological interventions.

## The Cutaneous Itch

### General Introduction

Itch is a common somatosensory modality well-known from the everyday life. It was defined as an “unpleasant sensation that elicits the desire or reflex to scratch” by Samuel Hafenreffer in the 17th century (Ikoma et al., 2006, p. 535), which is a pragmatic and valid definition even today. Our knowledge has been hugely expanded since Hafenreffer’s definition and, especially in the last 2 decades, we reached a deeper insight into the molecular and cellular details of how itch is generated, yet our understanding is far from complete. Although itch in general is not a life-threatening situation, the clinical management of itching conditions is still one of the biggest challenges of daily dermatological practice. Treatment of chronic itch (lasting longer than 6 weeks) remains an unmet medical challenge in many instances, affecting millions of people worldwide. According to epidemiological results the prevalence of chronic itch in the general population is between 8–28% (Weisshaar and Dalgard, 2009; Leader et al., 2015). Based on these data, it is not surprising that the socioeconomic burden of chronic pruritus is comparable to that of chronic pain. Development of effective treatments is mainly impaired by our lack of understanding of the signaling pathways underlying pruriception, especially in chronic itch, where itch develops and is maintained (at least partly) independently of external stimuli.

Itch can be classified into four categories based on both the different mechanisms by which it may be generated, and by taking into account their clinical appearence (Paus et al., 2006; Bíró et al., 2007; Ständer et al., 2007; Tóth et al., 2015; Dong and Dong, 2018). **Pruriceptive itch** is peripherally induced itch generated in the skin. In this case, itch is evoked by locally released pruritogens exciting the pruriceptive nerve endings in the skin. The release of these chemical mediators can be triggered acutely by external irritants (e.g. insect bite, poisonous plants or skin sensitizers) or may be related to various, typically inflammatory skin conditions which can affect an extended area of the skin, and is likely to be chronic. **Neurogenic itch** is also evoked by the (peripheral) excitation of itch sensitive neurons, but in this case the triggering pruritic mediators stem from a “central source” and their production is related to systemic diseases, such as kidney failure, hepatic conditions or immunological diseases. In contrast, **neuropathic itch** is due to a damage of the itch processing neural network at any level. It can be associated with peripheral neuropathies (e.g. postherpetic neuropathy), nerve compression or irritation (e.g. in notalgia paresthetica) or certain brain lesions and tumors. Finally, **psychogenic itch** is related to psychiatric disorders or psychological conditions like phobias, obsessive-compulsive disorder or psychotic diseases. In this review, we focus on the role of peripheral interactions in the generation of pruritus, therefore, mainly discussing cases of pruriceptive and some neurogenic pruritus as these are evoked by the excitation of pruriceptive cutaneous nerve endings.

### Sensing Pruritus

#### Pruriceptive Fibers of the Skin

The sensory transduction of pruritus, i.e. how propagating action potentials are generated by pruritic stimuli, is realized by exciting a subpopulation of cutaneous bare nerve endings which also express molecular markers typical of nociceptors. Therefore, pruriceptive fibers are generally considered a subpopulation of nociceptors, the selective activation of which results in itch sensation. This is in contrast to a general activation of nociceptors that results in nociception and evokes pain. This is postulated as the selectivity theory of itch, a nowadays generally accepted description of the relation between pruriception and nociception (LaMotte et al., 2014). This is also supported by the findings that depletion of nociceptors by overdosing transient receptor potential 1 (TRPV1) agonists (Cavanaugh et al., 2009; Imamachi et al., 2009) or genetic ablation of TRPV1-lineage nociceptive neurons of the dorsal root ganglia (DRGs) resulted in a dramatic reduction of both nociception and pruriception (Mishra et al., 2011; Mishra and Hoon, 2013). However, intense efforts were taken to identify molecular markers of a “labelled line” for pruriception. Among primary sensory neurons of the DRGs, a few molecular markers were identified which are believed to be (more or less) specifically expressed by pruriceptive sensory neurons. For example, specific neurotransmitters can be released from the central terminal of the pruriceptive sensory neurons which may differentiate these neurons from the nociceptor population. The fact that the ablation of gastrin releasing peptide receptor expressing (GRPR+) neurons–or only the GRPR molecules from the spinal cord–strongly inhibited pruritogen evoked scratching behavior without affecting acute nociception suggested that gastrin releasing peptide (GRP) may be a neurotransmitter released selectively from itch sensitive sensory neurons of DRGs. GRP was indeed detected in peripheral sensory neurons (Sun and Chen, 2007; Barry et al., 2016, 2020) and its expression was found to be elevated in chronic itch conditions in mice (Zhao et al., 2013) and primates (Nattkemper et al., 2013). The optogenic activation of GRP expressing cutaneous sensory fibers resulted in itch behavior, and chemically induced itch was attenuated by conditional deletion of GRP from DRG neurons (Barry et al., 2020). However, other studies resulted in controversial findings as they could not (or hardly) detect GRP in peripheral sensory neurons, rather localized it in the spinal cord, expressed by higher order neurons in the itch pathway (Fleming et al., 2012; Mishra and Hoon, 2013; Sun et al., 2017). Other studies argue for the role of natriuretic polypeptide b (NPPB) as a peripheral itch specific neurotransmitter. It was shown to be expressed in a subpopulation of DRG neurons and its genetic deletion, as well as ablating its receptor, dramatically decreased scratching behavior induced by various pruritogens (Mishra and Hoon, 2013). Moreover, members of Mas1-related G protein-coupled receptors (MRGPRs) were also identified as markers of itch specific neurons (Liu et al., 2009; Liu and Dong, 2015). In mouse, MRGPRs are coded by an extended gene cluster and divided into several subfamilies. However, in human, there are only four members of the family identified, marked as MRGPRX1-4, which do not form orthologous pairs with rodent counterparts (Dong et al., 2001; Lembo et al., 2002). Some MRGPRs, expressed exclusively in skin innervating fibers, are not only markers of pruriceptive neurons, but also serve as receptors for pruritic ligands. Especially the role of MRGPRA3, MRGPRC11 and MRGPRD, as well as the human MRGPRX1 were described to be involved in various forms of non-histaminergic itch. Interestingly, MRGPRA3 and MRGPRD display non-overlapping expression in pruriceptive neurons and are activated by different pruritic ligands, suggesting the existence of different labeled lines even within the non-histaminergic itch sensing neuron population (Liu et al., 2009, Liu et al., 2012 Q.; Han et al., 2013; Liu and Dong, 2015).

Recently, large scale transcriptome profiling studies also characterized and classified somatosensory neurons in an unbiased manner and identified different neuronal subpopulations potentially responsible for pruriception based on their specific expression patterns. Following single cell RNA-Seq, Usoskin et al. (2015) identified 11 clusters of somatosensory neurons by principal component analysis. Among them, itch associated markers (*Mrgpr*s, *Nppb*, histamine receptors (*Hr*s) serotonin receptors (*Htr*s) endothelin receptor A (*Ednra*), etc.) were highly and selectively enriched in the NP1-3 clusters which represents a fraction of the unmyelinated, small size neurons expressing the classical markers of non-peptidergic sensory neurons. Interestingly, within these clusters, marked inhomogeneity was found in the expression of particular pruritic markers, e. g. *Nppb* and IL-31 receptor (*Il31ra*) were highly expressed in the NP3 cluster, or *Mrgpra3* and *Mrgprd* displayed highest expression in different clusters, in line with previous data from “biased” studies. Clustering somatosensory neurons using a similar approach, Chiu et al. (2014) also identified a specific subset of DRG neurons highly expressing *Nppb* and *Il31ra* genes within the *Trpv1* ^*+*^ *Nav1.8/1.9* ^*+*^ nociceptor population. Interestingly, these cells were mainly negative for isolectin B4 (IB4), a classical marker of non-peptidergic nociceptors (Priestley, 2009). Most recently, a similar single neuron RNAseq transcriptome profiling identified that the neuronal clusters described in mice are highly conserved in non-human primates (Kupari et al., 2021). The NP1-3 classes were also identified in rhesus macaque expressing, among others, *MRGPRX1-4* in NP1-NP2 clusters and *HR1* in NP3. However, there are some remarkable interspecies differences in the expression of individual genes within some clusters. For example, although somatostatine (*SST*), Janus kinase 1 (*JAK1*), *IL31RA*, Oncostatin M receptor β (*OSMRβ*)*,* and Sphingosine-1-phosphate receptor 1 (*S1PR1*) were highly expressed in the NP3 cluster in both mice and macaque, *Nppb,* serotonin receptor 1F (*HTR1F*), and neurotensin (*NTS*) were specifically expressed only in mice whereas the expression of some other genes were mainly restricted to primates (Kupari et al., 2021). In a current study, Nguyen et al. (2021) classified human DRG neurons based on single nucleus RNA sequencing and supported their analysis with multiplex *in situ* hybridization. They grouped the sensory neurons into 15 classes (H1-H15) that mainly matched the previously described mouse clusters, but they also identified some human-specific classes which does not have a clear mouse counterpart. From the point of view of itch, H10 and H11 classes seem to be the most relevant. The expression pattern of these classes resembled to the mouse NP1-3 classes, likely representing non-peptidergic pruriceptive neurons. Neurons in the H11 class highly expressed *OSMRβ*, *JAK1* and *SST* especially similarly to NP3 mouse neurons, whereas *MRGPRX1* was found mainly in H10 as its counterpart genes are characteristic for NP2 mouse cells. However, both human classes also expressed markers characteristic for NP1 group in mice, and in general, the *in situ* hybridization indicated that H10 and H11 are relatively heterogeneous classes of sensory neurons. Interestingly, some H10 neurons co-expressed the low-threshold mechanosensitive ion channel *PIEZO2* with pruriceptive markers that was not found in mice. Thus, it is tempting to consider these cells as the mediators of the human mechanically evoked itch (Nguyen et al., 2021). It is important to mention that these data revealed remarkable differences in the expression of growth factor receptors between the corresponding neuron classes of mice and human, suggesting that the development and differentiation of the analogue somatosensory neurons might be controlled by different mechanisms in rodent and human (Nguyen et al., 2021).

Phenomenologically, pruriceptive fibers can be characterized by their (electro)physiological properties in humans (Schmelz, 2015). The unmyelinated C fibers innervating the skin contains mechanosensitive polymodal nociceptors responding to mechanical, chemical and thermal stimuli, and less numerous mechano-insensitive nociceptive fibers, as well (Schmidt et al., 1995, 1997, 2002). In this latter, mechano-insensitive group, a subset of neurons are identified by their marked responses to the prototypic itch mediator histamine, suggesting that they form an “itch-sensitive” population within the primary afferents. These histamine-sensitive sensory neurons were characterized by low conduction velocity, high transcutaneous electrical threshold, large receptive field and poor two point discrimination threshold for histamine-induced itch (Wahlgren and Ekblom, 1996; Schmelz et al., 1997, 2000, 2003; Schmidt et al., 2002; Schmelz and Schmidt, 2010). A distinct group of histamine insensitive pruriceptive afferents was also proposed by the experiments demonstrating that low intensity-high frequency focal electrical stimulation evoked itch sensation without causing erythema, which erythema is a characteristic consequence of the axon reflex activated by exciting the histamine sensitive fibers (Ikoma et al., 2005; Steinhoff et al., 2006). In contrast to histamine, the pruritic spicules of the cowhage (*Mucuna pruriens*) pod activated a subgroup of mechanosensitive nociceptive afferents and not the mechano-insensitive ones (Namer et al., 2008). Moreover, the involvement of nociceptive, myelinated A-fibers was also demonstrated in the itch sensation evoked by cowhage (Ringkamp et al., 2011).

These human data are in good accordance to the above mentioned rodent results describing distinct sub-groups of pruriceptive fibers within the nociceptor population. Indeed, results of rodent behavior experiments on pruriception can be successfully translated to human itch sensation, especially with the use of advanced experimental paradigms which are able to discriminate between nociception and pruriception in mice, like the cheek test or calf injection model. In the cheek model, compounds are injected into the cheek of the animals which results in wiping with the forelimb or scratching with the hind limb in case of algogens and pruritogens, respectively. Similarly, calf injection resulting in pain and itch will induce selectively licking and biting responses, respectively (Shimada and LaMotte, 2008; LaMotte et al., 2011, 2014). These techniques were found to be very useful to discriminate between pruriception and nociception and identifying the selective molecular events in the sensory transduction on pruritus.

#### Mechanisms of the Sensory Transduction in Pruritus

The activation of the above detailed pruriceptive primary sensory neurons is responsible for the sensory transduction of itch, which is the first step of pruriception, (i.e. the neural processing of the information which finally will result in itch sensation). The pruritic sensory transduction is typically initiated by chemical mediators acting on their receptors expressed by the cutaneous sensory terminals. During the molecular events of the sensory transduction of itch, pruritic mediators typically bind to a metabotropic receptor which initiates the activation of intracellular signaling pathways resulting in the opening of some ion channels responsible for the generator potential which finally evokes the discharge of the neuron (Tóth et al., 2015, 2020; Dong and Dong, 2018).

The ion channels involved in the initial depolarization are considered as molecular integrators and amplifiers of pruriception. The best studied of these ion channels mostly belong to the transient receptor potential (TRP) family of ion channels and show significant overlap with those involved in nociception. The pruriceptive role of the thermosensitive nociceptors TRPV1 and transient receptor potential ankyrin 1 (TRPA1) is the most characterized on sensory neurons (Wilson and Bautista, 2014, 201; Schmelz, 2015; Tóth et al., 2015, 2020). The role of TRPV1 was described primarily in the transduction of histamine induced pruritus, but it is involved in some forms of non-histaminergic pruritus as well (Imamachi et al., 2009; Dong and Dong, 2018). In contrast, TRPA1 is a general integrator in the transduction of itch induced by various non-histaminergic mediators (Wilson et al., 2011; Wilson et al., 2013; Lieu et al., 2014; Wilson and Bautista, 2014). Although histaminergic and non-histaminergic forms of pruritus signal via different pathways and may be transmitted by selective labeled lines, the partially overlapping expression of TRPV1 and TRPA1 is more widespread in sensory afferents and is not restricted to pruriceptors. Beyond their role in pruriception, they are thermosensitive and can mediate different forms of nociception as well: e.g. TRPV1 is a central molecule of inflammatory warm hyperalgesia and TRPA1 plays a role in cold and mechanical hyperalgesia (Tominaga et al., 1998; Caterina et al., 2000; da Costa et al., 2010; Julius, 2013; Vriens et al., 2014). However, in certain cases, TRPA1 and TRPV1 can play a synergistic role in the same process. They were recently shown as key transducers of heat-pain together with transient receptor potential melastatin 3 (TRPM3), another thermosensitive nociceptor TRP channel significantly co-expressed with TRPV1 and TRPA1. Interestingly, despite their co-expression and functional overlap in thermal nociception (Vandewauw et al., 2018; Held and Tóth, 2021), TRPM3 is not involved in transduction of pruritus evoked by either histamine or serotonin (5-HT) and endothelin-1 (ET-1) (Kelemen et al., 2021). These data suggests, that individual TRP channels, even if coexpressed by some sensory neurons, can play selective roles in certain forms of pruriception or nociception. Even different sensations evoked by the same substance can be mediated by different TRP channels: Sphingosine 1-phosphate (S1P) activates S1P receptor 3 (S1PR3) which induces both itch and pain. However, itch transduction is due to activation of TRPA1 *via* G_βγ_ signaling pathway but pain transduction realized by TRPV1 activation *via* PLC mediated signal transduction (Hill et al., 2018).

Beyond TRPV1 and TRPA1, other ion channels can integrate the effect of pruritogens. Recently, TRPV4 was described to mediate (at least some forms of) 5-HT evoked itch and cellular responses of DRG neurons (Akiyama et al., 2016). Beyond TRP channels, the Ca^2+^-activated chloride channel anoctamine 1 (ANO1/TMEM16A) (Yang et al., 2008) was proposed to mediate the activation of C fibers by chloroquine, a strongly pruritic antimalarial drug activating MrgprA3 (Ru et al., 2017). Similar to TRP channels, ANO1 is a thermosensitive nociceptor, as well: it can be activated by noxious warm temperature and mediates nociceptive responses in thermal pain models. Although ANO1 is a chloride channel, its activation can result in depolarization and consequent discharge of DRG neurons due to their relatively higher intracellular Cl^−^ concentration in physiological circumstances and it can contribute to the neural depolarization induced by ET-1 and histamine (Cho et al., 2012). Interestingly ANO1 can also amplify the neural activity elicited by depolarizing nociceptive Ca^2+^-permeable cationic channels. In nociceptors, Ca^2+^ influx via TRPV1 was demonstrated to activate ANO1, strongly exacerbating TRPV1 induced depolarization and nociception (Takayama et al., 2015, 2019). Similarly, TRPV4 can be also coupled to ANO1 as reported in secretory cells (Takayama et al., 2014; Derouiche et al., 2018).

#### Receptors for Pruritogens in Sensory Fibers

The activation of the above listed neuronal ion channels can be initiated by several receptors of the cutaneous pruriceptive nerve endings which are sensitive for the peripherally released pruritic mediators (Table 1). The most well-known, “traditional” pruritogenic mediator histamine binds to its G protein coupled ***histamine receptors*** (H1R, H3R, and H4R) that are linked to pruritus and expressed on the cutaneous sensory fibers (Panula et al., 2015) (Rossbach et al., 2011). Of these, activation of H1R and H4R excites pruriceptors resulting in itch. H1R signalizes via G_q/11_ proteins (Panula et al., 2015), and is shown to activate phospholipase C β3 (PLCβ3) and consequently TRPV1 (Han et al., 2006). Pharmacological data also support the involvement of phospholipase A2 and lipoxygenases in the H1R-induced activation of TRPV1 (Kim et al., 2004). H4R can also activate TRPV1 via a PLC-mediated pathway (Jian et al., 2016). Moreover, the role of protein kinase C δ (PKCδ) was also described in the activation of pruriceptors by histamine but not by non-histaminergic pruritogens (Valtcheva et al., 2015). In contrast, the activation of H3R, which is known as an inhibitory histamine receptor transmitting negative feedback on histamine release (Panula et al., 2015), seems to inhibit histamine-induced pruritic responses as its inverse agonists can evoke both activation of pruriceptive neurons and itch (Rossbach et al., 2011).

**TABLE 1:** Cutaneous pruritic mediators stimulating sensory nerve endings

Pruritic mediator	Potential sources	Potential targets on sensory fibers: Receptors, signal transduction, cellular responses
Histamine	Mast cells, basophils	H1R → G_q_ → PLCβ3/PLA2 → TRPV1
		H4R → PLC → TRPV1
		Further potential elements of transduction: PKCδ, lipoxygenase
Serotonin	Mast cells, keratinocytes	5-HTR7 → TRPA1
		TRPV4
Endothelin 1	Keratinocytes	ET_A_ → PLCβ3 → TRPA1
		Role of ERK1/2?
Proteases → BAM8-22, SLIGRL/SLIGKV	Mast cells, keratinocytes	(mouse) MRGPRC11 → G_αq_ → TRPA1
		(human) MRGPRX1/MRGPRX2
TSLP	Keratinocytes	IL7Rα-TSLPR → TRPA1
IL-31	Mast cells	IL-31RA-OSMRβ → ERK1/2 → TRPA1/TRPV1
IL-33	Keratinocytes	IL-1RAcP-ST2 → TRPA1/TRPV1
IL-13	T_h_2 type immune response	IL-13Rα1 → JAK1 → ?sensitization/activation?
IL-4	T_h_2 type immune response	IL-4Rα → JAK1 → ?sensitization/activation?
CXCL10	Neutrophils, Keratinocytes	CXCR3 → Ca^2+^-regulated Cl^−^ channels
LTC4	Various leukocytes, especially in T_h_2 type immune response	CysLT_2_R → TRPA1/TRPV1
S1P	Erythrocytes, Endothelial cells, Mast cells, Dendritic cells; increased in inflammation	S1PR3 → G_βγ_ → TRPA1 → itch (High concentration of S1P: S1PR3 → PLC → TRPV1 → pain)
Periostin	Keratinocytes, Fibroblasts	integrin α_V_β3 → TRPA1/TRPV1
(ds)RNA/hairpin structures of self-RNA	Tissue damage?	TLR3

5-HT is also a potent pruritogen in both humans and rodents (Weisshaar et al., 1997; Akiyama et al., 2010; Dong and Dong, 2018), which can activate several ***5-HT receptors*** expressed by sensory nerve endings. Among them, pharmacological activators of 5-HT_2A_ and 5-HT_7_ were shown to sensitize TRPV1 responses via PKC- and PKA-dependent pathways (Ohta et al., 2006). In contrast, the genetic ablation of the ionotropic 5-HT3A did not affect 5-HT evoked behavioral responses, while the lack of PLCβ3 diminished it. This further supports the role of the metabotropic 5-HT receptors and related signaling pathways in pruriception. However, genetic deletion of TRPV1 did not influence the 5-HT-induced responses which were mainly abolished in the absence of TRPV1 expressing sensory neurons arguing for the role of an alternative ion channel in the 5-HT signaling pathway in TRPV1+ neurons (Imamachi et al., 2009). Indeed, studies of gene deleted animals provided evidence for the role of 5-HT_7_ receptor in mediating acute serotonergic itch via consequent TRPA1 activation (Morita et al., 2015). Moreover, as mentioned above, TRPV4 was also described as a component in the transduction of serotonergic itch in sensory neurons (Akiyama et al., 2016).

The otherwise vasoconstrictive peptide ET-1 acts as an effective endogenously produced itch mediator, although ET-1 can also induce nociception (Hans et al., 2009; Gomes et al., 2012; Kido-Nakahara et al., 2014). Sensory neurons express mainly the ***ET receptor A*** (***ET***_***A***_) (Khodorova et al., 2009) which signals partly via a G_q_-related pathway stimulating PLCβ (Khodorova et al., 2009; Davenport et al., 2016). In nociceptors, ET_A_ activation by endothelin results in increase in intracellular Ca^2+^ concentration which activates PKC resulting in the potentiation of TRPV1 responses (Plant et al., 2007). The pruritic signaling also starts from ET_A_ (McQueen et al., 2007), and involves PLCβ3, but endothelin-evoked itch is independent of TRPV1 (Imamachi et al., 2009). It is strongly reduced by genetic deletion or pharmacological blockade of TRPA1 (Kido-Nakahara et al., 2014), although there is some controversy about the role of this ion channel in ET-1-induced scratching (Liang et al., 2011). Moreover, the role of extracellular signal-regulated kinases ERK1/2 was also proposed in the ET_A_ induced pruritic responses. ET-1-induced scratching is negatively regulated by the endothelin-converting enzyme 1 (ECE1) co-expressed with ET_A_ in somatosensory neurons (Kido-Nakahara et al., 2014).

As mentioned above, ***MRGPRs*** serve not only as markers of the pruriceptive fibers but also as receptors for some non-histaminergic pruritogens. As their name indicates, they are G protein-coupled receptors that signal mainly via the G_q_ pathway, resulting in Ca^2+^ release from intracellular stores. The deletion of a gene cluster of 12 *Mrgpr* genes in mice resulted in impaired itch evoked by selected non-histaminergic pruritogens: the antimalarial drug chloroquine was identified to activate MRGPRA3, and bovine adrenal medulla 8-22 (BAM8-22, a proenkephalin A-derived peptide) and SLIGRL (a peptide product cleaved from the protease activated receptor 2, PAR2) stimulate MRGPRC11. These receptors were shown to signal *via* G_αq11_ activating TRPA1 (and not TRPV1) (Lembo et al., 2002; Liu et al., 2009, 2011; Wilson et al., 2011; Liu and Dong, 2015). However, a recent study challenged the role of TRPA1 in mediating chloroquine-induced itch, and suggested the role of the calcium-activated chloride channel ANO1/TMEM16A as a downstream target of the chloroquine-induced PLCβ-mediated signaling resulting in depolarization and consequent discharge of sensory fibers (Ru et al., 2017). Importantly, these mouse Mrgpr ligands are also pruritogenic in humans, activating MRGPRX1 (chloroquine and BAM8-22) and MRGPRX2 (SLIGKV, the human analog of the SLIGRL) (Liu et al., 2009, 2011; Sikand et al., 2011; Liu and Dong, 2015). Moreover, in humans, MRGPRX4 also induces pruritic signal transduction *via* G_q_/PLC pathway upon activation by bile acids (Yu et al., 2019). Interestingly, in mice, bile acids activate another G-protein coupled receptor, the ***G-protein-coupled bile acid receptor 1*** (***TGR5***) in sensory afferents, which also signals via TRPA1 and evokes itch (Alemi et al., 2013; Lieu et al., 2014). Next to the above MRGPRs, the activation of MRGPRD by β-alanine can also induce itch. Similar to the previously mentioned receptors, MRGPRD activates TRPA1, albeit via a PKA-dependent manner (Liu Q. et al., 2012; Wang et al., 2019).

Although several cytokines, especially T_h_2-associated ones, are involved in the development of pruritus and itchy (dermatological) disorders, only some of them can directly excite pruriceptive nerve endings *via* ***cytokine receptors***. Neural cytokine receptors can influence the responsiveness of the sensory fibers even if their activation does not initiate immediate action potential firing. Therefore, they can contribute to the development of chronic itch characteristic of several of the most prevalent dermatological conditions (Storan et al., 2015). One of the most well-established pruritic cytokines is IL-31 which activates a subpopulation of sensory neurons via a *receptor heterodimer composed of IL-31 receptor A (IL-31RA) and Oncostatin M receptor β (OSMRβ)* (Cevikbas et al., 2014; Datsi et al., 2021). IL-31RA activation induces signal transduction through the activation of ERK1/2 and both TRPV1 and TRPA1 (Cevikbas et al., 2014). Sensory neurons also express receptors of other T_h_2-type cytokines, like IL-4 (*IL-4Rα*) and IL-13 (*IL-13Rα1*). Interestingly, although IL-4 and IL-13 also activate a small percentage of pruriceptive fibers in both mice and humans, they did not evoke acute itching, in contrast to IL-31. However, they sensitized the sensory neurons toward histamine and other pruritogens, and increased the intensity of histamine-evoked itch. These responses were mediated by IL-4Rα and downstream JAK1 signaling (Oetjen et al., 2017). A recent report raised some controversy about the itch-inducing effect of IL-4 and IL-13 demonstrating that they can evoke even acute itch if applied at lower concentration. A potential explanation might be that higher concentration of the cytokines saturates the JAK1 pathway, and induces negative feedback reactions (Campion et al., 2019). Sensory neurons also express the receptor of thymic stromal lymphopoietin (TSLP), which is another pruritic T_h_2-type cytokine produced by various epithelial cells, including epidermal keratinocytes (Wilson et al., 2013; Varricchi et al., 2018). It activates a small population of the cutaneous nerve endings expressing the heteromeric *TSLP receptor composed of IL7 receptor alpha (IL7Rα) and TSLP-specific receptor chain (TSLPR) chains*. The activation of TSLP receptor evokes itch via TRPA1 (Wilson et al., 2013). *IL-1 receptor accessory protein (IL-1RAcP) and a membrane-bound IL-33–specific ST2 form a heteromeric receptor* for IL-33, and both subunits can be found in the membrane of pruriceptive nerve endings. IL-33 is a pro-inflammatory cytokine, which can activate sensory neurons *via* ST2 receptor involving both TRPA1 and TRPV1. These IL-33-induced signaling pathways evoked itch in an urushiol-induced allergic contact dermatitis model (Liu et al., 2016; Topal et al., 2020). In early phase of AD and contact hypersensitivity model of allergic contact dermatitis, *CXC chemokine receptor 3 (CXCR3)* was found to be upregulated in pruriceptive neurons as was its ligand CXCL10 in the surrounding tissue. In these models, antagonist of CXCR3 inhibited spontaneous disease-related itch (Qu et al., 2015, 3; Walsh et al., 2019). Pharmacological evidence suggests that CXCR3 may signal via a Ca^2+^-regulated chloride channel (Qu et al., 2017).

***Toll-like receptors*** (***TLRs***) belong to pattern recognition receptors that are activated by exogenous pathogen- or endogenous danger-associated molecular patterns (PAMPs or DAMPs, respectively). Expressed in various immune cells and peripheral tissues, they are key players in initiating innate immune responses. Among them, TLR3 and TLR7 are expressed in sensory neurons and have been suggested to play roles in the development of pruritus (Taves and Ji, 2015; Dong and Dong, 2018). TLR3 activation by its ligand polyinosinic-polycytidylic acid (poly I:C) evoked action potential firing in sensory neurons and induced acute scratching behavior. Moreover, TLR3 was found to be important in the development of both histaminergic and non-histaminergic itch as both were markedly decreased in *Tlr3* knock out animals (Liu T. et al., 2012). Like TLR3, TLR7 was also detected in peripheral sensory neurons of the DRGs and TLR7 activators evoked acute itching in a TLR7-dependent manner. Moreover, the TLR7 agonist imiquimod induced discharge of DRG neurons in wild type, but not in *Tlr7* ^−/−^ mice (Liu et al., 2010). Although both TLR3 and TLR7 are mostly known to be localized in intracellular membranes, it is proposed that they can be expressed in the surface membrane of the sensory neurons, and are thereby available for extracellular ligands (Taves and Ji, 2015). However, the role of TLR7 was questioned by another study indicating that imiquimod-induced scratching as well as neuronal responses are independent of TLR7, but may be due to the inhibition of background or voltage-gated potassium channels of the somatosensory neurons (Kim et al., 2011; Lee et al., 2012). Moreover, imiquimod was recently shown to directly activate ***TRPA1*** which, in sensory neurons, may initiate immediate acute itch (Esancy et al., 2018; Kemény et al., 2018).

Recently, the extracellular matrix protein periostin was shown to activate the receptor ***integrin α***_***V***_ ***β3*** on the surface of DRG neurons resulting in itch behavior in mice. The periostin induced, integrin α_V_β3-dependent itch was strongly reduced in mice lacking TRPA1 and TRPV1 ion channels, and NPPB suggesting that these ion channels and neurotransmitters of the pruriceptive neurons are involved in the periostin evoked itch. However, the signaling pathway connecting the integrin α_V_β3 to TRP channels is still under investigation (Mishra et al., 2020; Hashimoto et al., 2021a).

As mentioned above, the activation of ***Sphingosine 1-phosphate receptor 3*** (***S1PR3***) by S1P can also initiate itch activating TRPA1 via G_βγ_ signal transduction. However, the same receptor can also activate TRPV1 *via* PLC dependent signaling but this pathway results in nociception and can only be activated by higher S1P concentration (Hill et al., 2018).

Somatosensory neurons also express ***Lysophosphatidic acid receptor 5*** (***LPA5***), a receptor for lysophosphatidic acid (LPA), that mediates LPA evoked itch (Kittaka et al., 2017; Yamanoi et al., 2019). In the LPA5 signaling pathway, LPA can be (re-)generated intracellularly mainly via phospholipase D (PLD), but PLA_2_ and PLC can be also involved. Finally, intracellular LPA can directly activate TRPV1 and TRPA1 resulting in the excitation of the pruriceptive neurons (Nieto-Posadas et al., 2011; Kittaka et al., 2017).

Currently, ***cysteinyl leukotriene receptor 2*** (***CysLT***_***2***_ ***R***), the receptor of the cysteinyl leukotriene C4 (LTC4), was described as a highly expressed receptor in sensory neurons. CysLT_2_R was detected especially in the subset of NP3 cluster DRG neurons, strongly coexpressed with *Il31ra* and *Nppb*. Its activation by LTC4 induced acute scratching behavior via CysLT_2_R. Although it was not investigated whether CysLT_2_R activates DRG neurons or not, and the downstream signaling pathway is also largely unknown, LTC4-induced scratching was diminished in *Trpv1* knock out mice and in the presence of TRPA1 antagonist suggesting that both ion channels can mediate the effect (Voisin et al., 2021).

***Urokinase plasminogen activator receptor*** (***U-PAR***) was also reported in a subset of DRG neurons and its agonist serpin E1 evoked Ca^2+^ transients in DRG neurons as well as itching in mice, but the mechanism of action and the related signaling pathway is largely unknown (Larkin et al., 2021).

### Cutaneous Pruritic Crosstalk

**FIGURE 1:**
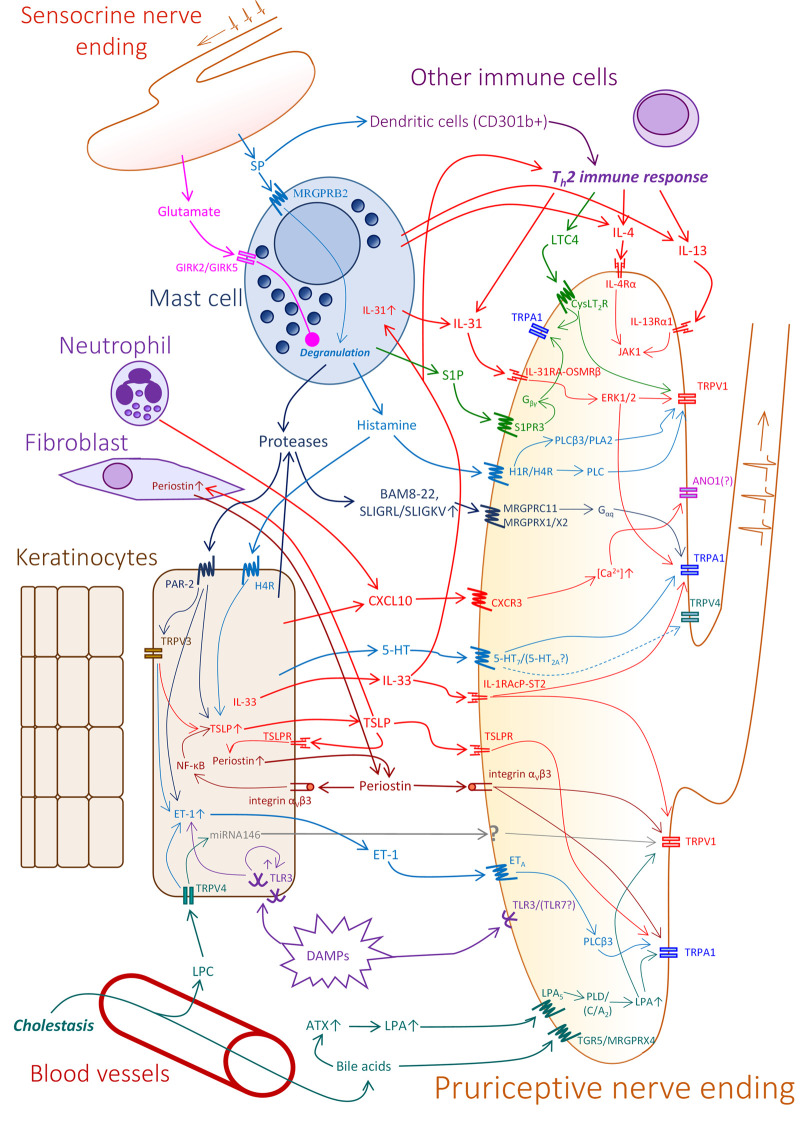
Potential elements and mechanisms in pruritic cutaneous crosstalk. In the skin, products of keratinocytes, mast cells, several immune cells, and additional metabolic and tissue factors can contribute to the excitation of pruriceptive sensory nerve endings. Moreover, sensory terminals can also release pro- and anti-pruritic factors. Note, that the sensory nerve ending in the figure represents a hypothetic pruriceptor demonstrating the expression of several receptors and signaling pathways which may be expressed by different individual sensory neurons. For more detailed explanation, please see the text. Abbreviations: *5-HT*–Serotonin, *5-HT*_*2A*_ */*_*7*_—Serotonin receptor_2A_/_7_, *ANO1*—Anoctamine 1, *ATX*–Autotaxin, *CXCL10*—C-X-C motif chemokine ligand 10, *CXCR3*—C-X-C Motif Chemokine Receptor 3, *DAMPs*—Damage associated molecular patterns, *CysLT2R*—cysteinyl leukotriene receptor 2, *ERK1/2*—Extracellular signal-regulated kinase 1/2, *ET-1*—Endothelin 1, *ET*_*A*_—Endothelin receptor A, *GIRK2/5*—glutamate ionotropic receptor kainate type subunit 2/5, *IL-13Rα1*—Interleukin 13 receptor, alpha 1, *IL-1RAcP-ST2*—IL-1 receptor accessory protein - ST2 heterodimer, *IL-31RA-OSMRβ*—IL-31 receptor A- Oncostatin M receptor β heterodimer, *IL-4Rα*—Interleukin 4 receptor, *JAK1*—Janus kinase 1, *LPA*—Lysophosphatidic acid, *LPA*_*5*_—Lysophosphatidic acid receptor 5, *LPC*—lysophosphatidylcholine, *LTC4*—cysteinyl leukotriene C4, *MRGPRB2/C11/X1/X2/X4*—Mas-related G-protein-coupled receptor B2/C11/X1/X2/X4, *NF-κB*—nuclear factor kappa-light-chain-enhancer of activated B cells, *PLD/C/Cβ3/A*_*2*_—phospholipase D/C/Cβ3/A_2_, *S1P* - Sphingosine 1-phosphate, *S1P3R*—Sphingosine 1-phosphate receptor 3, *SP*—Substance P, *TGR5*—G-protein-coupled bile acid receptor 1 (Takeda G protein-coupled receptor 5), *TLR3/7*—Toll-like receptor 3/7, *TRPA1/V1/V3/V4*—Transient receptor potential Ankyrin 1/Vanilloid 1/Vanilloid 3/Vanilloid 4, *TSLP*—Thymic stromal lymphopoietin, *TSLPR*—Thymic stromal lymphopoietin receptor.

Recent advances in the field have shown that itch does not necessarily start at the level of the nerves, but can also be initiated by non-neuronal elements in the skin. In this section we will list the possible contribution of cutaneous cells to the development of itch through mediator release (skin-nerve axis), as well as the role of factors released by nerves that act locally to propagate both the release of pruritogens and local inflammation (nerve-skin axis) (Figure 1).

#### Keratinocytes’ Contribution to Itch

Keratinocytes were classically considered to be important in forming and maintaining the skin barrier. This view has been supplemented by numerous observations, including the fact that keratinocytes express sensory receptors, among others TRP channels (Denda et al., 2001; Inoue et al., 2002; Peier et al., 2002; Bodó et al., 2004; Chung et al., 2004) and that they can actively secrete various substances that communicate with neighboring cells and nerve endings, including ATP (Denda and Denda, 2007; Mandadi et al., 2009; Mihara et al., 2011), dopamine (Fuziwara et al., 2005) and glutamate (Fuziwara et al., 2003). Based on these results keratinocytes can be considered as the forefront of the sensory nervous system (Denda et al., 2007).

In terms of itch sensation, the triggers that can elicit the release of pruritogens from keratinocytes are still not fully known. Keratinocytes express multiple receptors that have been implicated in itch induction, including PAR2 (Buhl et al., 2020), TLR3 (Szöllősi et al., 2019), H1 and H4 receptors (Gschwandtner et al., 2008; Schaper et al., 2016), ET_A_ and endothelin receptor B (ET_B_) (Tsuboi et al., 1995), 5-HT receptors (Lundeberg et al., 2002; Slominski et al., 2003), OSMRβ (Boniface et al., 2007; Kato et al., 2014), integrin α_V_β3 (Masuoka et al., 2012), TSLPR (Mishra et al., 2020), as well as neuropeptide receptors (Sandoval-Talamantes et al., 2020), and two members of the transient receptor potential vanilloid (TRPV) family, TRPV3 and 4 (Peier et al., 2002; Sokabe et al., 2010; Mihara et al., 2011; Tóth et al., 2014; Szöllősi et al., 2018). While we have evidence that the receptors listed above are all functionally expressed by keratinocytes, their role in the development and propagation of itch is less well-defined. In general we can classify them into two large groups: receptors that influence the barrier forming function of keratinocytes, and those that cause the cells to secrete factors that can activate pruritic nerve endings. The former may be considered as an indirect mechanism of itch induction, since impaired barrier function leads to increased transepidermal water loss, dry skin, and an increased likelihood of exogenous pruritogens penetrating the stratum corneum (Yosipovitch et al., 2019). The latter can be considered a direct mechanism of itch signaling, where the activated keratinocytes secrete signaling molecules (IL-33, TSLP and ET-1) known to activate pruritic nerve fibers.

The indirect path of itch induction as mentioned above is dependent on the disruption of the epidermal barrier. This is usually accompanied by the production of pro-inflammatory cytokines (e.g. IL-6) and chemokines (e.g. CXCL-8, CCL17/TARC, CCL19/MIP-3β, CCL22/MDC, CCL23/MIP-3, CCL4/MIP-1β and CXCL1/GRO1α) (Cornelissen et al., 2012; Kabashima, 2013; Lee et al., 2013), as well as nerve growth factor (NGF) by keratinocytes. The combined effect of these factors is recruitment of further inflammatory cells to the skin, and in the case of chronic pruritus, increased density of nerve fibers and response in the affected area (Buhl et al., 2020). The disruption of the epidermal barrier can occur through increased keratinocyte proliferation (as caused by agonists of H4R (Glatzer et al., 2013), TLR3 (Szöllősi et al., 2019), periostin (Masuoka et al., 2012), and neuropeptides (Sandoval-Talamantes et al., 2020)), through disruption of the differentiation process, by the production of matrix metallopeptidase 9 (MMP9), which can drive recruitment of immune cells to the skin [as caused by agonists to H1 (Gschwandtner et al., 2013; Chen J. et al., 2021)], and the increased production of antimicrobial peptides, a common characteristic of inflammatory skin diseases [as seen after OSMRβ activation (Boniface et al., 2007)]. TRPV3, a non-selective calcium-permeable channel first identified in keratinocytes (Peier et al., 2002), was linked to pruritus based on the consequence of gain-of-function mutations in the channel. These lead to the hairless and pruritic dermatitis phenotype of DS-*Nh* mice (Yoshioka et al., 2009) and to the development of Olmsted syndrome in humans, which is characterized by palmoplantar keratoderma and periorificial plaques, as well as hair and nail malformities, pain and itch (Lin et al., 2012). Conversely, the activation of TRPV4 accelerates barrier recovery, and the formation of intercellular junctions between keratinocytes since this channel is co-localized to adherent junction proteins such as E-cadherin and β-catenin. The role of TRPV4 in the development of itch is more nuanced however, since it has also been linked to regulation serotonin and histamine release as well as a consequent development of itch (Chen et al., 2016; Luo et al., 2018; Boudaka et al., 2020).

Of the above listed mediators, their involvement in direct keratinocyte-nerve communication has been proven for H4R (Schaper et al., 2016), PAR2 (Kempkes et al., 2014; Buhl et al., 2020) TLR3 (Szöllősi et al., 2019) and both TRPV3 (Seo et al., 2020) and TRPV4 (Moore et al., 2013). To date the major pruritic mediators that can directly activate pruritic nerve endings released by keratinocytes are TSLP, periostin, ET-1, IL-33 and most recently BNP. On keratinocytes histamine dominantly acts through the H4 receptor and increases the release of TSLP subsequently to poly I:C stimulation in both murine and human cells (Schaper et al., 2016). TSLP secretion has also been observed after activation of all the above mentioned receptors with the exception of TRPV4, which solidifies its role as one of the most important skin-derived pruritic mediators (Kinoshita et al., 2009; Wilson et al., 2013; Park et al., 2017). Furthermore, TSLP (and other T_h_2 cytokines) can induce periostin secretion and periostin can stimulate further TSLP release potentially establishing another pruritic positive feedback (Masuoka et al., 2012; Mishra et al., 2020; Hashimoto et al., 2021a). One of the most potent keratinocyte-derived pruritic mediators is ET-1 (Kido-Nakahara et al., 2014), the production of which can be initiated by PAR2 (Buhl et al., 2020), TLR3 (Szöllősi et al., 2019), TRPV3 (Zhao et al., 2020), and TRPV4 (Chen et al., 2016) activation. IL-33 is a member of the IL-1 inflammatory cytokine family, and is constitutively expressed in the nucleus of keratinocytes, and acts as an alarmin that is released upon inflammation or cellular damage (Moussion et al., 2008). While first shown to act on cells of the innate and adaptive arm of the immune system [specifically mast cells, type 2 innate lymphoid cells, basophils and type 2 T helper cells (Kondo et al., 2008)], the receptor of IL-33, ST2 is also expressed on sensory nerve endings in the skin and its activation leads to an itch response in mice, as discussed above (Liu et al., 2016). Moreover, IL-33 is upregulated in atopic dermatitis (AD) lesions which may contribute to its pruritic phenotype (Imai, 2019).

As seen above the contribution of keratinocytes to itch sensation is multifaceted, and this is further complicated by the interplay between these receptors in the keratinocytes themselves. PAR2 has been shown to signal through TRPV3 (Zhao et al., 2020), the expression of TLR3 is increased upon TLR3 activation (forming a positive feedback loop that could be a major factor in the chronification of itch (Szöllősi et al., 2019)), and the effect of histamine is also potentiated through TLR3 (Schaper et al., 2016). This is also compounded by the crosstalk between keratinocytes and immune cells, as detailed below.

#### Immune Cells

Mast cells have long been considered to be a central player in the pathogenesis of itch, mainly through their release of histamine. Histamine is the main mediator responsible for acute itch, by activating the H1 and H4 receptors on sensory nerves (Shim and Oh, 2008). Mast cells also release a wide array of other signaling molecules including cytokines and chemokines, and have recently been shown to contribute to non-histaminergic itch as well (Meixiong et al., 2019a) through these mediators. The direct role of mast cells in itch transduction is further supported by their close proximity to afferent nerves in the skin (Bienenstock et al., 1991). The most important cytokines known to directly activate pruritogenic nerves are IL-4, IL-13 and IL-31, all of which are associated with T_h_2 cells and which can be released from mast cells, among others. Other sources of these cytokines include natural killer cells, basophils and eosinophils, although the contribution of these latter cell populations to itch is not well defined.

The role of T_h_2 cells in pruritic skin diseases, especially in AD, is well documented (Gandhi et al., 2017), and forms the basis for some of the most effective treatments of itch in AD (Ruzicka et al., 2017; Gooderham et al., 2018; Bawany et al., 2020). Dupilumab inhibits the effect of IL-4 and IL-13 by blocking the IL-4α subunit which is shared by both cytokines, while Nemolizumab targets IL-31RA to block the effect of IL-31 (Ruzicka et al., 2017). IL-4 and IL-13 act both as amplifiers of T_h_2 responses that contribute to the upkeep of the environment that promotes pruritic signaling (Furue et al., 2019), and as direct activators of pruritic nerve endings (Campion et al., 2019). IL-31 acts on both keratinocytes and sensory nerves (Sonkoly et al., 2006), although its direct link to itch is only proven in mice, since in humans it does not induce immediate, only delayed itch responses (Hawro et al., 2014). Nevertheless, Nemolizumab has been proven to be efficacious in the treatment of AD (Ruzicka et al., 2017), which hints that in humans IL-31 acts indirectly to induce itch.

An indirect contribution of the abovementioned cytokines to the development of chronic itch is their contribution to barrier dysfunction, by the downregulation of skin barrier proteins in keratinocytes (Kim et al., 2008). Since this may lead to the release of TSLP that strengthens T_h_2 cell functions (Kitajima et al., 2011), we can once again see a possible positive feedback loop that may lead to the chronification of itch. Keratinocytes may also produce IL-33, which also leads to T_h_2 polarization in AD (Imai, 2019), and results in IL-31 secretion from mast cells (Petra et al., 2018). Recently, next to its direct pruritogenic effect, the role of the extracellular matrix protein periostin emerged as a regulator of barrier functions, and an amplifier of T_h_2 responses, as well (Hashimoto et al., 2021a).

#### Neurogenic Pruritus – Role of Sensory Neurons in the Establishment of a Pruritus-Prone Local Milieu

The crucial role of the cutaneous immune cells and keratinocytes in the establishment of a local pruritic environment via the secretion of mediators that signal toward the itch-detecting sensory fibers in various skin conditions is unquestionable. However, emerging evidence supports the concept that this is not a one-way interaction, but rather a local pruritic intercellular network between the sensory neurons and the peripheral cells, in which the neurons can also actively take part by influencing the function of the neighboring cells, and thereby contributing to the development of an inflamed, pro-pruritic local tissue micromilieu. The “classical” concept of neurogenic inflammation has been known since the ‘60s, when Miklós Jancsó and his colleagues showed that the excitation of capsaicin-sensitive nerve endings causes local inflammation (Jancsó et al., 1967, 1968). Later research described the “sensocrine” function of the sensory neurons by which they release neuropeptides such as substance P (SP) and calcitonin gene related peptide (CGRP), as well as glutamate, ATP, chemokine (C–C motif) ligand 2 (CCL2), colony stimulating factor 1 (CSF-1) and other mediators, including even micro-RNAs at the peripheral nerve endings. These sensory neuron-derived mediators can influence the local barrier and immune functions as well as inflammatory responses (Shouman and Benarroch, 2021). As time passes, more and more specific “neuron-to-periphery” interactions are identified, and some of them are likely to have an impact on the development of pruritus.

Neuropeptides released from the sensory nerve endings, especially from C-fibers, can target keratinocytes, dermal endothelial cells, mast cells, Langerhans cells, and lymphocytes as well. For example, SP can increase histamine and TNFα release from mast cells, IL-1, IL-6 and IL-8 production in keratinocytes, or IL-8 production in dermal microvascular endothelial cells, all contributing to local inflammation (Ansel et al., 1997; Choi and Di Nardo, 2018; Shouman and Benarroch, 2021). In contrast to the inflammatory role of SP, the effect of CGRP is more ambiguous. It can activate mast cells, evoke vasodilation (Ansel et al., 1997; Choi and Di Nardo, 2018; Shouman and Benarroch, 2021), and shift Langerhans cells-initiated immune responses toward T_h_2 direction (Ding et al., 2008), but it was also found to inhibit 5-HT- or histamine-induced inflammatory responses (Granstein et al., 2015), as well as the T_h_2 cytokine production in type 2 innate lymphoid cells (Wallrapp et al., 2019). Somatostatin is also released from the peripheral sensory endings, but it evokes rather anti-inflammatory responses (Szolcsányi et al., 1998; Helyes et al., 2004, 2009).

As described above, T_h_2 cell mediated immune responses play a crucial role in the development and maintenance of a pruritic tissue environment. It was shown in a mouse AD model that substance P-dependent neurogenic inflammation mediated the stress-evoked shift in the cutaneous cytokine profile toward T_h_2 cytokines (Pavlovic et al., 2008). Moreover, sensory neuron-derived SP can regulate allergic responses as well. It was recently described that allergen house dust mite proteases activate TRPV1 expressing sensory neurons resulting in SP release, and SP then induces mast cell degranulation *via* MRGPRB2 (Serhan et al., 2019). Another recent study also demonstrated that intradermal injection of protease allergens initiated not only immediate itch and pain behavior, but stimulated SP (and inhibited CGRP) release from TRPV1 expressing sensory fibers. The released SP activated the CD301b + dendritic cells and induced their migration to draining lymph nodes, where these cells were responsible for T_h_2-differentiation. It was found that ablation or pharmacological blockade of allergen responder TRPV1+ sensory neurons decreased allergen-induced T_h_2 cell differentiation and related IL-4 and IL-13 expression (Perner et al., 2020). Moreover, CGRP released from cutaneous sensory nerve endings was also described to stimulate CD301b+ dendritic cells to produce IL-23 in murine skin, which resulted in increased IL-17A production of γδT cells (Kashem et al., 2015).

Importantly, cutaneous sensory neurons can not only initiate but also suppress this inflammatory, pruritic environment. In contrast to neuropeptides, some non-peptidergic fibers expressing MRGPRD can negatively regulate mast cells *via* glutamate release, which likely acts via an ionotropic glutamate receptor heterodimer composed of glutamate ionotropic receptor kainate type subunit 2 (GIRK2/GLUR6) and GIRK5 expressed by mast cells (Zhang et al., 2021).

## Pruritic Diseases

Pruritus is a common symptom of several dermatological and systemic diseases with the involvement of the above discussed cutaneous immuno-neuronal crosstalk. In the next part of our review, we provide a concise summary of some of these diseases and the potential mechanisms which can lead to the development of the pruritic symptoms (Table 2).

**TABLE 2:** Overview of the potential pathogenesis of itch in selected pruritic diseases and pathological conditions.

*Disease*	Factors potentially involved in the pathogenesis of pruritus
Irritant contact dermatitis (ICD)	Keratinocyte injury and barrier damage → inflammatory response, T_h_1 cytokines
Allergic contact dermatitis (ACD)	Allergen specific, T cell mediated inflammatory responses, typically T_h_2 type → 5-HT↑, ET-1↑, TSLP↑, CXCL10↑, IL-33↑
Urticaria	IgE, degranulation of mast cells, dysregulation of basophils and eosinophils → histamine↑, other pruritic mediators
Atopic dermatitis (AD)	Barrier disturbances, vicious itch-scratch cycle → irritant and allergen permeation↑
	Type 2 inflammation: IL-4↑, IL-13↑, IL-31↑, TSLP↑
	Dysregulation of cutaneous signaling pathways: opioid, cannabinoid, neuropepitide (SP→NKR1) signaling
	Innervation density↑
	Inflammatory lipid mediators↑
	Periostin synthesis↑ (linked to type 2 inflammation)
Psoriasis	T_h_17↑ → IL-17↑, IL22↑
	IL31↑, TSLP↑, SP↑, NPY↓
	NGF↑ → innervation density↑
Prurigo nodularis	Innervation density↑ → SP↑, CGRP↑
	Eosinophils↑, mast cells↑, T cells↑ → IL4↑, VIP↑, histamine↑ prostaglandins↑
Cutaneous T-cell lymphoma	IL-31↑, IL-31RA↑, OSMRβ↑
	IL-4?, IL-13?, SP?
Dermatomyositis	CD4^+^ cells↑ → IL-31↑, IL-31RA↑
Systemic sclerosis	Neuropathic component: Destruction of sensory fibers by accumulating collagen, and later regeneration by the inflammatory milieu
	Mast cells↑, histamine↑
Chronic renal failure	Eosinophils↑, mast cells↑, histamine↑, tryptase↑, inflammation↑
	Peripheral neuropathy
	Imbalance of μ- and κ -opioid receptor activity
Cholestatic liver diseases	Endogenous opioids↑, histamine↑, serotonin↑, lysophosphatidic acid↑ (→TRPV1), bilirubin↑, bile acids↑
	Lysophosphatidylcholine → TRPV4 (epidermis) → miR-146a↑ → neural TRPV1 activation↑
	Bile acids↑ → TGR5 → TRPA1 (mouse)
	Bile acids↑ → MRGPRX4 (human)
	BAM8-22↑ → MRGPRX1/MRGPRC11↑ → TRPA1

### Dermatological Diseases

#### Contact Dermatitis

There are two forms of contact dermatitis to distinguish: irritant and allergic contact dermatitis (ICD and ACD, respectively). In case of ICD, the primary cause is a (chronic) exposure to irritants that causes epidermal barrier perturbation. Epidermal keratinocytes are the primary targets of the irritants. Upon exposure, they subsequently synthesize and release pro-inflammatory cytokines that are not biased toward T_h_2-mediated immune responses. Indeed, the infiltrating T cells rather belong to the T_h_1 class and especially IL-2, IFNγ, IL-1α, IL-1β, IL-6, CXCL-8, TNFα, GMCSF, and VEGF are upregulated in the skin (Lisby and Baadsgaard, 2006; Lee et al., 2013). Importantly, different irritants can differentially affect cytokine levels, and can evoke various biological responses in keratinocytes ranging from hyperproliferation to necrotic cell death. Itch is a common symptom in ICD, but pain-like sensations (e.g., stinging or burning) are also often observed (Lisby and Baadsgaard, 2006; Bains et al., 2019). In contrast, ACD is a delayed, type 4 hypersensitivity reaction, i.e., an antigen-specific T-cell mediated inflammatory response to repeated exposure. It is composed of two distinct immunological phases, i.e., the sensitization and the elicitation or effector phase. In the sensitization phase, the haptens penetrate the epidermal barrier, and establish direct contacts with various skin components including MHC molecules expressed by epidermal Langerhans cells resulting in the activation of said cells. Activated, allergen-presenting Langerhans cells travel to the draining lymph nodes to be recognized by specific T cells. This process is associated with a cascade of cytokine production that stimulates the proliferation of the specific allergen-recognizing T cells which finally enter the circulation in high numbers. Upon a repeated allergen contact, the effector phase is initiated. The activated antigen presenting cells recruit the circulating primed T cells that will locally be activated by the allergens and will subsequently release large amounts of inflammatory cytokines thereby contributing to the local inflammation. This reaction typically peaks in 12–48 h after the allergen exposure. This process involves both type 1 and type 2 cytokines, which, beyond the inflammatory responses, can initiate ACD-associated sensory phenomena, like itch (Rustemeyer et al., 2006; Leonard and Guttman-Yassky, 2019).

A recent study compared itch and pain behavior, and scored the accompanying inflammation in mouse hypersensitivity models of ICD and ACD. The authors applied the same topical challenge for both conditions, using the hapten squaric acid dibutylester (SADBE; challenge with 1% solution on three consecutive days). The two experimental protocols only differed in the sensitization phase, when SADBE was applied to the abdominal skin of the animals belonging to the ACD group, whereas ICD mice as well as members of the control group received acetone. Finally, both group was challenged with 1% SADBE on the tested skin area (cheek or calf). It was found, that both ICD and ACD are characterized by itch- and pain-related behavior, inflammatory symptoms and pro-inflammatory cytokine production, but in general, both the sensory symptoms as well as the inflammatory features are stronger in ACD than in ICD. However, C-X-C motif chemokine ligand 10 (CXCL10) was elevated only in ACD (Zhang et al., 2019). Moreover, in SADBE-induced murine ACD, chemokine receptor CXCR3, which serves as the receptor of CXCL10, was upregulated in the DRG and its pharmacological inhibition attenuated spontaneous itch, but not pain. Injected CXCL10, on the other hand, evoked itch-, but not pain-related behavior in ACD mice (Qu et al., 2015).

Importantly, the exact pathomechanism, the cytokine production, and T-cell polarization in contact dermatitis largely depend on the allergen both in humans and in mice (Dhingra et al., 2014; Leonard and Guttman-Yassky, 2019). For example, nickel induces T_h_1-biased responses with certain T_h_17 and T_h_22 elements, whereas house dust mite elicits T_h_2-polarized responses with additional T_h_9 and T_h_17/T_h_22 activation, and fragrance allergens cause T_h_2/T_h_22 skewed immune responses (Dhingra et al., 2014; Malik et al., 2017; Leonard and Guttman-Yassky, 2019). Allergen-specific reactions were also identified in skin biopsies after food patch tests applied in delayed-type hypersensitivity food reaction. For example, besides T_h_17 polarization, peanut, but not beef or codfish, was also characterized by increased IL-33 expression (Ungar et al., 2017; Leonard and Guttman-Yassky, 2019). In mouse pruritic ACD models, the generally used oxazolone induced a mixed T_h_1/T_h_2 response with elevated level of 5-HT, ET-1, and substance P, but not TSLP. In contrast, the poison ivy-driven allergen urushiol resulted in T_h_2-biased responses associated with increased IL-33, TSLP, 5-HT, and ET-1 expression without affecting substance P (Liu et al., 2016, 2019). These mediators are capable of activating their own receptors in the itch-sensitive sensory neurons that transduce the pruritic signals likely via TRPA1 in oxazolone- and urushiol-induced ACD (Liu et al., 2013).

#### Urticaria

Chronic urticaria (CU) is characterized by the occurrence of weals (hives), angioedema, or both for more than 6 weeks, and is usually accompanied by severe pruritus (Gonçalo et al., 2021). It is estimated to affect ∼1% of the population, and it may significantly impair quality of life (Gonçalo et al., 2021). Although the signaling pathways involved in the development of itch in CU are not completely explored, pathological degranulation of dermal mast cells as well as dysregulation of basophil and eosinophil granulocytes and the subsequent histamine release appear to be central players in the process (Hon et al., 2021).

Indeed, the classical symptoms of urticaria are well-modelled by intradermal histamine injection. Besides pruritus, locally applied histamine also causes increased vascular permeability and development of edema (weal), as well as local vasodilation resulting in dermal hyperaemia (erythema), which latest is a consequence of neuropeptide release (SP and CGRP) from the activated mechano-insensitive, peptidergic C fibers. These symptoms are specifically related to histamine and are not associated to non-histaminergic itch (Andersen et al., 2015). Although urticaria is mainly characterized by a histaminergic nature, clinical data indicate that both histaminergic and non-histaminergic components may be involved in the development of pruritus in CU. Indeed, second generation antihistamines are recommended to be the first choice to alleviate itch (Hon et al., 2019), and, if they remain ineffective in spite of the elevation of their dose, administration of IgE-neutralizing antibodies (e.g., Omalizumab or the more effective Ligelizumab) (Wedi, 2020), corticosteroids, leukotriene receptor antagonists (e.g., montelukast), Cyclosporine A, or even certain antidepressants and anti-inflammatory drugs should be considered (Hon et al., 2019).

Importantly, recent research suggests that other mast cell and basophil granulocyte-related targets may also become useful tools in the treatment of CU. These include spleen tyrosine kinase (SYK; a down-stream target of the high-affinity IgE receptor FcεR1α), Bruton tyrosine kinase (BTK; an important regulator of IgE-independent mast cell activation), CRTh2 (a receptor for PGD2 expressed among others on mast cells), as well as H4 histamine and MRGPRX2 receptors (Hon et al., 2021). Last, but not least, besides the aforementioned biological drugs targeting mostly mast cells and basophils, eosinophil-targeting [e.g., ones neutralizing IL-5 [mepolizumab and reslizumab] or IL-5 receptor (benralizumab)], and other antibodies (interfering with the signaling of IL-1, IL-4, and TNF-α) also showed promising effects in clinical trials [for details, see (Hon et al., 2021)].

#### Atopic Dermatitis

AD is a chronic, inflammatory skin disease affecting ca. 20% of children and ca. 10% of adults in the industrial countries (Langan et al., 2020). Based on the symptoms and certain aspects of the pathogenesis, it can be classified into two major subtypes, i.e., “extrinsic” and “intrinsic” AD (Czarnowicki et al., 2019). The extrinsic endotype is more common. It usually develops on an atopic background, and it is characterized by eosinophilia, high serum IgE level, and greater filaggrin mutation rate as compared to the intrinsic endotype that exhibits female predominance, delayed onset, as well as lack of atopic background, and is characterized by a relatively more preserved barrier function, normal serum IgE level, and an increased prevalence of metal contact hypersensitivity (Czarnowicki et al., 2019). Importantly, the existence of intra-endotype variations in the immune polarization and epidermal barrier function is also well-described across different races (Czarnowicki et al., 2019). However, despite of the aforementioned complexity, it is well-evidenced that disturbance of each element of the complex cutaneous barrier (i.e., physicochemical, immunological and microbiological) (Proksch et al., 2008; Jensen and Proksch, 2009) is a key contributor in the development of AD (Bieber, 2008; Griffiths et al., 2017). Although clinical symptoms of the disease may exhibit a great inter-individual heterogeneity (Langan et al., 2020), development of eczematous lesions, intense pruritus, and a chronic or relapsing disease course are characteristic features of AD.

Of great importance, itch is not only one of the most unpleasant symptoms of AD, but, *via* the “itch-scratch cycle”, it also contributes to the pathogenesis of the disease by damaging the epidermal barrier, and facilitating the permeation of allergens and irritants (Mack and Kim, 2018; Furue et al., 2020; Langan et al., 2020; Nakahara et al., 2021). Thus, alleviating itch could be much more than a mere symptomatic treatment in AD. Research efforts of the last decades have highlighted the role of several itch mediators and pathways in AD-related pruritus (Langan et al., 2020; Umehara et al., 2021). Indeed, periostin (Mishra et al., 2020) and type 2 cytokines (e.g., IL-4, IL-13, IL-31, and TSLP) (Yang and Kim, 2019) and most especially, the IL-4—neuronal IL-4 receptor interaction, together with the subsequent JAK-1 signaling appear to be key players in the process (Cevikbas et al., 2014; Oetjen et al., 2017), whereas histamine and its key pruritic receptors are likely to be of inferior significance (Umehara et al., 2021). Indeed, “classical” anti-histamines targeting H1R failed to be effective as “add-on” therapy in eczema (Matterne et al., 2019), although recently, promising results were published with H4R antagonists (Werfel et al., 2019) and combination of H1R and H4R antagonists (Köchling et al., 2017). On the other hand, dupilumab (a human monoclonal antibody blocking the effects of IL-4 and IL-13 and thereby interfering with the activation of T_h_2 cells and group 2 innate lymphoid cells (Beck et al., 2014; Imai et al., 2021)) monotherapy was greatly efficient in reducing Eczema Area and Severity Index (EASI) and pruritus scores in a double-blind placebo-controlled trial involving patients with moderate-to-severe AD (Beck et al., 2014). Likewise, nemolizumab (CIM331), a humanized antibody against interleukin-31 receptor A could also significantly improve pruritus in patients with moderate-to-severe AD in a phase 2, randomized, double-blind, placebo-controlled study (Ruzicka et al., 2017).

Recent pieces of evidence argue that dysregulation of other signaling pathways (e.g., cutaneous cannabinoid (Tóth et al., 2019), and opioidergic signaling (Slominski, 2015; Bigliardi et al., 2016)) may also contribute to AD-related pruritus. Indeed, CB_1_ and CB_2_ cannabinoid receptors, as well as κ-opioid receptor (KOR) were found to be significantly downregulated in the lesional skin of AD patients suffering from severe itch as compared to the non-itchy, non-lesional skin of the patients (RNAseq) (Nattkemper et al., 2018). In line with these data, KOR, as well as dynorphin A 1-17 and dynorphin A 1-8 were found to be down-regulated in the lesional epidermis of AD patients (Tominaga et al., 2007), and topically applied nalfurafine (a selective KOR agonist) alleviated itch in AD (Inui, 2015; Elliott et al., 2016). Moreover, the serum concentration of β-endorphin (an endogenous opioid exhibiting higher affinity towards the “pro-pruritic” μ-opioid receptor) was found to be elevated in AD, and its level correlated with the severity of itch (Lee et al., 2006). Taken together, these pieces of evidence suggest that dysregulation of homeostatic cutaneous cannabinoid and opioid signaling may contribute to the development of pruritus in AD.

Importantly, several other peptide and lipid signaling pathways were also suggested to be involved in the development of AD-related itch. Indeed, both the number of mast cell–sensory nerve contacts, as well as the number of SP and CGRP positive nerve fibers were elevated in the lesional epidermis of AD patients as compared to healthy controls (Järvikallio et al., 2003). Moreover, the NKR1 antagonist aprepitant was found to exert significant anti-pruritic effects in AD suggesting that the SP–NKR1 pathway is also important in AD-related itch (Ständer et al., 2010). Moreover, 12/15-LOX and COX pathways were also found to be dysregulated in the lesional skin of AD patients leading to the elevation of the levels of several potentially pruritogenic lipid mediators, including 12-hydroxy-eicosatetraenoic acid (12-HETE), leukotriene B4 (LTB4), thromboxane B2 (TXB2), prostaglandin (PG) E2, and PGF2 (Töröcsik et al., 2019).

Last, but not least, it should also be noted that TRPV3 is a potent promoter of the production and release of various pro-inflammatory regulators on multiple cell types of the human skin, including keratinocytes and sebocytes (Szöllősi et al., 2018; Szántó et al., 2019), and is likely to play a role in dry skin dermatoses (Szántó et al., 2019), including AD as well as AD-related pruritus. Indeed, PAR2 and TRPV3 were shown to be up-regulated in skin biopsies of AD patients (Zhao et al., 2020). Activation of PAR2 on epidermal keratinocytes was shown to influence Ca^2+^-homeostasis of the cells *via* STIM1-Orai1 interaction, resulting in TSLP release leading to itch (Wilson et al., 2013). More recently, it has also been demonstrated that the ability of keratinocyte PAR2 activation to evoke TSLP release and subsequent itch can be abrogated by the genetic deletion of TRPV3, arguing that the two receptors may cooperate in mediating itch in AD (Zhao et al., 2020). Finally, according to a recent study, the IL-31-induced BNP-release from the sensory neurons increases TRPV3 expression and activity on epidermal keratinocytes in a natriuretic peptide receptor 1 (NPR1)-dependent manner. Enhanced activity of TRPV3 in turn led to elevated SERPIN E1 [a.k.a. plasminogen activator inhibitor 1; an adipokine expressed in keratinocytes as well (Kovács et al., 2020)] release that evoked itch (Larkin et al., 2021). Thus, abrogation of TRPV3 activity promises to be a powerful tool to alleviate itch in AD.

#### Psoriasis Vulgaris

Psoriasis is a common inflammatory skin disease affecting 1–3% of the world population (Szepietowski and Reich, 2016) characterized by sharply demarcated, erythematous, pruritic plaques covered in grey scales that can cover large areas on the extensor surfaces of the limbs, the trunk, and the scalp (Rendon and Schäkel, 2019). Even though the name derives from the Greek word for itch (psora), pruritus has long been an overlooked aspect of the disease, even though 60–90% of psoriatic patients report itch as one of their symptoms. Indeed, many report pruritus as the most bothersome of their symptoms (Komiya et al., 2020). The treatment of pruritus in psoriatic disease poses an unmet need, since antihistamines are generally considered to have only moderate effects, and the exact cause of itch remains unknown in psoriatic lesions (Domagała et al., 2017).

Psoriasis can easily be considered an immuno-epithelial disease, since the main driving factor of plaque development is the production of IL-17 and IL-22 by T_h_17 cells, which is initiated by TNFα and IL-23 from dendritic cells (Zheng et al., 2007). These cytokines have not been linked directly to pruritus, and it is likely that itch develops as a secondary consequence of the disease, instead of being a primary symptom that leads to the development of the psoriatic plaques. Nevertheless monoclonal antibody treatment targeting IL-17 has been reported to improve itch in psoriasis (Bushmakin et al., 2015; Strober et al., 2016; Kimball et al., 2018).

The role of multiple pruritic mediators have been investigated in psoriasis, including neuropeptides, nerve growth factor, IL-31, and TSLP (Komiya et al., 2020). Neuropeptides, specifically SP and Neuropeptide Y (NPY), have been implicated in the pruritus found in psoriasis. The levels of SP (Saraceno et al., 2006), as well as the number of SP + fibers in pruritic psoriatic lesions (Nakamura et al., 2003) is increased, and serlopitant, an antagonist of the neurokinin-1 receptor, was effective against chronic itch (Yosipovitch et al., 2018). Interestingly, the effect of SP on murine dorsal root ganglia was found to be more dependent on MrgprA1 (Azimi et al., 2017), so it is possible that in humans SP also acts on MRGPRX2, which is also highly expressed in psoriatic lesions (Nattkemper et al., 2018). NPY, on the other hand is found at lower levels in psoriatic patients with pruritus (Reich et al., 2007), which is possibly explained by the finding that it suppresses mechanical itch transmission in wild-type mice (Acton et al., 2019). NGF expression was also found to be higher in psoriatic lesions, as well as the expression of the NGF receptor tropomyosin-receptor A, both of which correlated with the intensity of pruritus (Nakamura et al., 2003).

Multiple lines of evidence support the role of two classically AD-linked cytokines, TSLP and IL-31 in psoriasis. The serum level of both TSLP and IL-31 is elevated in patients with pruritic psoriasis (Narbutt et al., 2013; Suwarsa et al., 2019), as well the number of IL-31-immunoreactive mast cells at lesional sites (Niyonsaba et al., 2010), while TSLP expression is increased in the epidermis of psoriatic lesions (Volpe et al., 2014). TSLP has also been linked more directly to scalp psoriasis (Volpe et al., 2014).

The sphingolipid metabolite S1P is also associated to psoriasis. S1P, similar to IL-23, primes the maturation of T_h_17 cells via S1PR1 (Huang et al., 2007; Liao et al., 2007). Indeed, in psoriasis, elevated plasma S1P level was reported (Checa et al., 2015; Myśliwiec et al., 2017), which may stimulate pruriceptive fibers via S1PR3 (Hill et al., 2018), as discussed above.

The involvement of the nervous system in the pathogenesis of the disease has been suspected for some time. Multiple reports (Raychaudhuri and Farber, 1993; Zhu et al., 2016; Onderdijk et al., 2017; Keçici et al., 2018; Qin et al., 2021) showed that denervation of the skin on one side of the body can lead to the clearance of the lesions, and that stress can exacerbate the disease (Harvima et al., 1993; Singh et al., 1999). The mechanisms behind these observations were considered to be both increased local production of neuropeptides (Hosoi et al., 1993), and changes in the density of innervation in psoriatic lesions. Interestingly, both increased (Naukkarinen et al., 1991) and decreased innervation have been reported (Pergolizzi et al., 1998), as well as some reports that found no significant differences (Di Francesco et al., 1978; Armagni et al., 1979). Since, as mentioned above epidermal keratinocytes in psoriasis produce increased levels of NGF (Pincelli, 2000), it is logical to assume that this would influence the growth of nerves (Kou et al., 2012). Applying a selective optogenetic stimulation of TRPV1+ cutaneous nerve endings in mice resulted in the development of type 17 inflammatory response associated with histological features that highly resembled the imiquimod-induced psoriasiform lesions. The ablation of TRPV1+ sensory fibers attenuated these responses clearly indicating that psoriasiform symptoms can develop on neurogenic inflammatory background (Cohen et al., 2019).

#### Prurigo Nodularis

Prurigo nodularis is a chronic inflammatory skin disease characterized by multiple extremely pruritic lesions commonly found on the trunk and the extensor surfaces of the extremities (Mullins et al., 2021). Prurigo nodularis commonly occurs with other diseases, including AD, xerosis cutis, excoriation disorder, hypertension, type II diabetes mellitus, chronic kidney disease, HIV infection, substance-use disorders, mood disorders, and obesity (Mullins et al., 2021; Pereira et al., 2021). The exact pathophysiology of the disease is still unknown, but a strong neural component is likely based on increased number of protein gene product 9.5 immunoreactive nerve fibers and increased expression of SP and CGRP in the lesions (Abadía Molina et al., 1992; Lee and Shumack, 2005). Both neuropeptides stimulate local immune responses and promote endothelial cell proliferation through the release of vascular endothelial growth factor, and further increase in the number of nerve fibers through NGF production (Choi and Di Nardo, 2018). In terms of the immune system, lesional skin in PN contains a dense infiltrate of eosinophils, mast cells and T cells. These cells contribute multiple cytokines to the inflammatory milieu of the lesions, including IL-4 and VIP from eosinophils (who can also contribute NGF and SP) (Johansson et al., 2000), IL-31 from T lymphocytes and macrophages (Hashimoto et al., 2021b) and histamine and prostaglandins from mast cells (Zeidler et al., 2018).

#### Cutaneous T-Cell Lymphoma

Cutaneous T-cell lymphoma is typically divided into two common subtypes: mycosis fungoides (MF) and its leukemic variant, Sézary syndrome (SS). Pruritus affects a large population (approximately 88%) of both subtypes, and the severity of itch increases in late-stage disease, as well as being higher in general in SS (Vij and Duvic, 2012; Nattkemper et al., 2016). Pruritus in these patients responds poorly to treatment, which is unsurprising considering the fact the exact mechanism behind it is still unknown. Various mediators have implicated, although mostly based on empirical experience in a limited number of patients.

IL-31 levels are higher in sera of patients with MF and SS (Ohmatsu et al., 2012; Malek et al., 2015), although other results show contradictory results (Möbs et al., 2015). It is also unclear whether the IL-31 serum levels correlate with disease or pruritus severity (Malek et al., 2015). IL-31 expression locally in the skin is also increased, as well as the level of IL-31RA and OSMRβ (Nattkemper et al., 2016).

IL-4 and IL-13 may also play important roles in itch in these patients, since dupilimab significantly reduced itch in a patient with SS (Steck et al., 2020). SP may also be involved in itch in both SS and MF, since multiple reports show that the NK_1_ receptor antagonist aprepritant showed some efficacy in alleviating pruritus (Duval and Dubertret, 2009; Booken et al., 2011; Ladizinski et al., 2012; Torres et al., 2012; Song et al., 2017).

### Selected Systemic Diseases and Pathological Conditions

#### Dermatomyositis

*Dermatomyositis (DM)* is a rare heterogeneous systemic autoimmune connective tissue disease, which is a subtype of idiopathic inflammatory myopathies. DM might have a wide variety of clinical manifestations including lung, joint, esophageal and cardiac findings; however, its hallmark features are the characteristic skin manifestations and progressive symmetrical muscle weakness (Griger et al., 2017). Based on data from question surveys, the majority of the patients (84.6–90.4%) suffers from pruritus (Shirani et al., 2004; Kim et al., 2018) which has a significant impact on quality of life (QoL). It seems that the severity of pruritus significantly correlates with disease activity, but the pathogenesis of DM-related itch is poorly understood. One small retrospective study (Khanna et al., 2020) found that there is a trend between histopathologic presence of eosinophils in skin biopsies, and pruritus. Recently, it was shown that IL-31 and IL-31RA gene expression in lesional skin was upregulated compared with either non-lesional skin or that from healthy controls (Kim et al., 2018). IL-31 mRNA expression also positively correlated with itch score and immunoreactivity for IL-31 and IL-31RA was greater in lesional skin. Furthermore, lesional DM skin contained significantly more IL-31-producing cells, of which CD4^+^ cells were the most abundant IL-31-expressing cell type (Kim et al., 2018). Importantly, lenabasum (a.k.a. JBT-101 or ajulemic acid), an investigational, non-psychotropic, orally bioavailable CB_2_ receptor agonist with remarkable anti-inflammatory potential, was recently found to significantly downregulate IL-31 in CpG-stimulated PBMCs *in vitro* (Kim et al., 2018). These data indicate that lenabasum may have potential as a new therapy for DM and DM-related itch; its efficiency is also currently being tested in a phase 3 clinical trial (NCT03813160).

#### Systemic Sclerosis

*Systemic sclerosis (SSc)* is a rare and complex chronic autoimmune connective tissue disease characterized by Raynaud’s phenomenon (RP), nailfold capillary changes, excessive production of collagen, manifested as skin thickening and fibrosis of internal organs such as the lungs, heart, gastrointestinal tract, and kidneys. Clinically, patients can be subdivided into limited cutaneous SSc (lcSSc) and diffuse cutaneous SSc (dcSSc) forms. Pruritus represents a common, but infrequently reported skin symptom in SSc, with a prevalence of 43–62% (El-Baalbaki et al., 2010; Razykov et al., 2013; Théréné et al., 2017). It is significantly associated with greater skin and gastrointestinal involvement (Razykov et al., 2013) as well as worse mental status, physical function, and disability (El-Baalbaki et al., 2010).

The pathophysiology of pruritus in patients with SSc is unknown. Pruritus could arise either from 1) abnormalities of nerve fiber endings in SSc skin, 2) from the presence of an excess of mast cells, and/or 3) from the release of local mediators in the skin. The role of chemokines (derived not only from mast cells, but possibly also from other perivascular immune cells) on SSc itch is also of interest (Frech and Baron, 2013). Previous clinical observations in SSc, namely that pruritus is associated with sensory symptoms that predominate in the extremities and non-sclerotic areas, argue for a neuropathic component of pruritus (Théréné et al., 2017). The compression of small nerve fibers by thickened and/or dense collagen might contribute to the neuropathic component. A reduction of sensory and autonomic innervation in both sclerotic and apparently uninvolved skin has been reported in SSc (Provitera et al., 2005) with mast cell association early in the pathologic process. In addition, an inflammatory and immunological component of neuropathic pruritus had also been adduced by regeneration of C-fibers after destruction by collagen deposition and increasing sensitization of itch fibers by inflammatory mediators (Haber et al., 2016). On the other hand, elevated levels of histamine were found in 56% of patients with SSc and was more common in patients with diffuse disease (74%), in contrast to limited disease (31%), thus mast cells and histamine could also be the part of the pathogenesis of SSc-related itch (Falanga et al., 1990).

#### Chronic Renal Failure

*Chronic renal failure* is a worldwide public health problem with a global prevalence rate of 13.4% (Hill et al., 2016). Based on data of a multicenter study from 18,801 hemodialysis patients, moderate to extreme pruritus was experienced by 42% (Pisoni et al., 2006). The importance of pruritus is underlined not only because of poorer quality of life of the patients, but pruritus in HD patients was significantly associated with a 17% higher mortality risk (Pisoni et al., 2006). Although the association between chronic renal failure and skin itching has been recognized for more than a century, the exact pathogenesis of pruritus in renal failure remains poorly understood. It seems that uremic pruritus cannot be caused by a single factor, whereas many metabolic factors have been implicated in the pathogenesis of itching. Based on the existing literature, 4 general theories have emerged: 1) toxin deposition, 2) peripheral neuropathy, 3) immune system dysregulation, and 4) opioid imbalance (Verduzco and Shirazian, 2020).

The initial theory of chronic renal failure associated pruritus pathogenesis is deposition of pruritogenic toxins in the skin, like uremic compounds, vitamin A, calcium, phosphorus, and magnesium. This theory is mainly based on early observations, like pruritus is associated with underdialysis, improvement of pruritus after treatment of high calcium, parathormone and phosphorus level (Hampers et al., 1968; Hiroshige et al., 1995; Kimata et al., 2014; Mettang and Kremer, 2015), but further studies have not confirmed these associations (Shirazian et al., 2013). It is currently thought that underdialysis and toxin deposition may cause pruritus in a subset of patients, which should resolve with adequate dialysis. The second theory is based on the fact that in patients with chronic dialysis, there is a high prevalence of peripheral neuropathy and autonomic dysfunction, which could lead to abnormal skin innervation and nerve conduction resulting itch sensation. The pruritus in uremic patients occurred significantly more frequently in patients with paresthesia (Verduzco and Shirazian, 2020) and gabapentin, a drug approved for the treatment of epilepsy and neuropathic pain significantly reduced pruritus score, supporting the neuropathic hypothesis of uremic pruritus (Gunal et al., 2004).

The elevated levels of pro-inflammatory cytokines and inflammatory markers (C-reactive protein, IL-6, and IL-2) in patients with severe pruritus argue for the hypothesis that dysregulation of the immune system underlies pruritus in kidney disease (Kimmel et al., 2006; Narita et al., 2006). It is further supported by the fact that after kidney transplantation patients do not complain about pruritus as long as immunosuppressive therapy, including cyclosporine or tacrolimus is administered, even when a substantial loss of transplant function has occurred (Mettang et al., 2002). In addition, in small randomized trials thalidomide and tacrolimus ointment were effective in the therapy of uremic pruritus (Silva et al., 1994; Pauli-Magnus et al., 2000). However the effectivity of this latter compound was not proved by another vehicle controlled study (Duque et al., 2005). Increased levels of eosinophils, mast cells, histamine and tryptase have also been found in patients with uremic pruritus, which might draw attention to this hypothesis (Francos et al., 1991; Dugas-Breit et al., 2005).

Finally, the alteration of the opioid pathway, modulation of its receptors and cellular processes might also affect chronic renal failure associated pruritus. Overstimulation of central μ-opioid receptors, antagonism of peripheral κ-opioid receptors, or an imbalance of stimulation and antagonism of μ- and κ -opioid receptors causes itching (Tey and Yosipovitch, 2011; Verduzco and Shirazian, 2020). In a recently published double blind, randomized trial, difelikefalin, a peripherally restricted and selective agonist of κ opioid receptors, had a significant reduction in itch intensity and improved itch-related quality of life as compared with those who received placebo in patients undergoing hemodialysis who had moderate-to-severe pruritus (Fishbane et al., 2020). In addition, nalfurafine hydrochloride, an κ-opioid-selective agonist, has been officially approved for resistant pruritus in HD patients on the basis of a well-evidenced clinical trial in Japan (Inui, 2015).

#### Cholestatic Liver Diseases

Pruritus is a common and debilitating symptom in patients with *cholestatic liver pathology* such as cholangiocellular (primary biliary cholangitis, primary sclerosing cholangitis), obstructive biliary (gallstone diseases, pancreatic head carcinoma, etc.,) and hepatocellular (pregnancy related cholestasis, viral hepatitis) diseases. The pathogenesis of itch in cholestatic disease remains elusive and is likely multifactorial, while the mechanisms of HCV-associated pruritus are attributed to HCV induced cholestasis and the induction of interferon stimulated genes as a result of viral overload (Alhmada et al., 2017). Numerous candidate pruritogens are present in bile and upregulated in cholestatic patients, including endogenous opioids, histamine, serotonin, lysophosphatidic acid (LPA), bilirubin, and bile acids (BA) (Beuers et al., 2014). These substances might interact with the nerve endings of the skin and induce the sensation of itching. One of these key factors is Autotaxin (ATX), whose elevated activity in patients’ sera may be a consequence of HCV-induced chronic liver diseases and has a crucial role in the synthesis of LPA (Ikeda et al., 2009). Under pathological conditions elevated bile salts in patient’s tissues and sera are able to signal the activation of ATX-LPA signaling (Nguyen et al., 2014), leading to the accumulation of LPA, which can initiate the pruritus via its receptor LPA_5_ (Yamanoi et al., 2019). LPA is able to trigger the activation of TRPV1 and TRPA1 (Nieto-Posadas et al., 2011; Kittaka et al., 2017) and regulation of LPA by PI3k, protein kinase A (PKA) and C (PKC)-dependent mechanisms has been reported (Kassmann et al., 2013). Most recently, the LPA precursor lysophosphatidylcholine (LPC) was shown to directly bind to and activate epidermal TRPV4 resulting in the release of a micro RNA (miR-146a) which activates pruriceptive cutaneous nerve endings eliciting itch. The miR-146 evoked neural activation depended on TRPV1 activation, although the exact mechanism is not discovered yet. Importantly from a clinical point of view, elevated levels of LPC and miR-146 were found in pruritic primary biliary cholangitis patients compared to non-itching patients and the concentration of both LPC and miR-146a correlated with the severity of itch, although in another sample, there was no significant difference between primary biliary cholangitis patients and healthy subjects (Chen Y. et al., 2021).

Furthermore, animal experiments revealed that BAs activate TGR5 on sensory nerves. TGR5 is a G_s_ protein coupled bile acid receptor that is known to regulate metabolic processes in several tissues (Guo et al., 2016), which stimulates the release of neuropeptides in the spinal cord that transmit itch and analgesia (Alemi et al., 2013). It was found that TRPA1 is involved in this BA-evoked, TGR5-dependent pruritus in mice (Lieu et al., 2014). However, recently, experiments based on expression characterizations as well as functional assays revealed that TGR5 is not expressed in human DRG neurons and does not directly mediate itch sensation in human (Yu et al., 2019). Instead, it was proved, using humanized mouse models and calcium imaging, that the human sensory neuron-expressed MRGPRX4 is a novel bile acid receptor. The data suggest that targeting MRGPRX4 is a promising strategy for alleviating cholestatic itch (Meixiong et al., 2019b; Yu et al., 2019). Moreover, in a mouse biliary duct ligation model of cholestasis, that induced spontaneous itching, an increased expression of BAM8-22 was detected in the skin, and its receptor MRGPRX1/MRGPRC11 and the related TRPA1 were also upregulated in the sensory neurons of the DRGs. In this model, the number of the BAM8-22 responsive neurons was increased and BAM8-22 seemed to potentiate the spontaneous itching behavior suggesting that peripheral BAM8-22 may also mediate cholestatic itch (Sanjel et al., 2019). However, the recent recommendations for the treatment of cholestasis associated pruritus are based on 1) ursodeoxycholic acid (UDCA), which enhances biliary secretion of bile salts and other cholephiles; 2) the non-absorbable anion exchange resin cholestyramine, which binds a wide variety of amphiphilic molecules in the small intestinal lumen, increasing the bile excretion; 3) the potent pregnane X receptor agonist rifampicin, altering the metabolism of the presumed pruritogens in the liver and/or the gut by biotransformation and 4) opioid antagonists such as naltrexone and serotonin reuptake inhibitors such as sertraline, affecting the endogenous opioidergic and serotonergic system (European Association for the Study of the Liver, 2009; Bergasa, 2011; Kremer et al., 2011; Beuers et al., 2014).

## Therapeutic Conclusions

No universal itch-specific therapy has been established to date. The European guideline on chronic pruritus recommends several therapeutic options including diseases specific and various sympthomatic interventions based on a careful diagnosis and several individual factors (Weisshaar et al., 2019). The application of these diverse clinical approaches in different diseases and increasing evidence from recent research suggest that the anti-pruritic philosophers’ stone probably does not even exist. Inefficiency of antihistamines is well-established in several cases, and often-used immunosuppressants, for example glucocorticoids or calcineurin inhibitors are far from being specific in targeting pruritus or any specific pruritic disorders. They evoke their beneficial effect by generally suppressing immune responses, including several local immune-neuronal interactions characteristic for individual pathologies (Buddenkotte and Steinhoff, 2010). Specifically targeting this intercellular crosstalk locally in the skin may provide a highly specific and effective therapeutic approach with fewer side effects. The possible targets can be the pruritic mediators, their receptors and the related signaling pathways carefully chosen in light of the underlying pathology. The putative efficiency of such a selective approach is supported by several successful attempts using monoclonal neutralizing antibodies targeting T_h_2 and T_h_17 cytokines or their receptors e.g., in the treatment of AD and psoriasis, respectively (Fourzali et al., 2020; Zhang and He, 2020; Garcovich et al., 2021). The related signal transduction of these receptors can also be successfully targeted e.g., by JAK or phosphodiesterase 4 inhibitors (Fourzali et al., 2020; Soeberdt et al., 2020). Future alternative targets may be neural receptors (e.g., MRGPRs), signaling molecules, and ion channels (e.g., TRP channels) involved in the transduction of itch (Buddenkotte and Steinhoff, 2010; Tóth et al., 2015; Moore et al., 2018; Xie and Hu, 2018; Golpanian and Yosipovitch, 2020). However, the investigation of the specificity of these novel methods requires a cautious approach since certain molecular elements may overlap with different sensory transduction systems. For example, TRPV1 and TRPA1 are known not only as pruritic ion channels, but they also take part in temperature sensation and regulation, as well as in nociception (Vriens et al., 2014; Moore et al., 2018). Therefore, better understanding of the cutaneous pruritic interactions and identification of novel therapeutic targets are of great importance in current pruritus research.
